# Haptic Training Simulator Use in Emergency Procedure Education

**DOI:** 10.7759/cureus.105722

**Published:** 2026-03-23

**Authors:** Salil D Phadnis, Collin Hickey, Scott M Alter, Lisa M Clayton, Joshua J Solano, Richard D Shih, Robert Levine, Patrick G Hughes

**Affiliations:** 1 Department of Emergency Medicine, Florida Atlantic University Charles E. Schmidt College of Medicine, Boca Raton, USA; 2 Department of Emergency Medicine, Washington University in St. Louis, St. Louis, USA; 3 Department of Emergency Medicine, Kaiser Permanente Baldwin Park Medical Center, Baldwin Park, USA; 4 Department of Emergency Medicine, ArchieMD, Inc., Boca Raton, USA; 5 Department of Emergency Medicine, The University of Utah, Salt Lake City, USA

**Keywords:** emergency procedure, haptic trainer, lateral canthotomy, needle thoracostomy, virtual reality

## Abstract

Background: Although simulation-based education is integral to emergency procedure training, computer monitor screen-based simulation (SBS) alone cannot develop the requisite feel and psychomotor skills. Haptic training simulations providing force feedback via a multi-degree-of-freedom stylus may improve this issue, offering tactile realism without the resource burdens of high-fidelity mannequins and consumable task trainers. This study compared user experience and skill acquisition between traditional task-trainer-based simulation and SBS with haptic feedback.

Methods: We conducted a crossover study with 22 emergency medicine learners (residents and medical students) to compare haptic-augmented SBS (3D Systems Touch™ with custom Unity™ software; 3D Systems, Inc., Rock Hill, SC, USA; Unity Technologies, San Francisco, CA, USA) versus traditional physical task trainers for lateral canthotomy (LC) and needle decompression (ND) procedures. Participants completed pre- and post-knowledge tests (five multiple-choice items per procedure) and post-simulation Likert surveys (1-5 scale) assessing realism, usability, and educational value. Knowledge changes were analyzed using the paired-samples proportion, Newcombe's method, and McNemar's test; Likert responses were summarized using median and interquartile range.

Results: Mean knowledge scores for LC increased by 20.9% (p < 0.001), and haptic simulation achieved median Likert ratings of 4-5 for realism, ease of use, and desirability. ND knowledge improved in certain topics (needle gauge choice, p = 0.007) with median Likert ratings of 4-5 for utility and integration potential.

Conclusions: Haptic simulation represents a feasible and effective adjunct for procedural training in emergency medicine. For complex, dexterity-intensive tasks, such as LC, haptic augmented simulators offer educational outcomes comparable to those of traditional physical models while providing significant logistical and cost advantages. Conversely, traditional trainers remain at least as effective for less complex procedures such as ND. These preliminary findings support the integration of haptics into medical curricula, although future research must focus on long-term skill retention and the optimization of simulator design across a broader range of clinical interventions.

## Introduction

Simulation-based training has grown increasingly prevalent in medical and professional education, with computer monitor and virtual reality headset screen-based simulation (SBS) providing readily accessible instruction for cognitive and decision-making skills. However, a limitation of SBS is its inability to develop technical skills requiring physical interaction due to its virtual nature. As a result, procedural skill acquisition often relies on physical task trainers and high-fidelity mannequins, which, despite their effectiveness, demand substantial resources in comparison to their virtual counterparts [1,2]. In particular, mannequins are expensive consumables requiring a well-equipped staff and dedicated space for proper maintenance and operation [3].

Haptic trainers aim to address SBS’s shortcomings by providing an interface that more closely mimics the actions of a particular procedure compared to a standard mouse and keyboard. One subset of trainers resembles a motorized stylus that measures movement in multiple degrees of freedom and provides force feedback, allowing a user to manipulate and receive tactile feedback from three-dimensional on-screen objects. These have gained popularity in surgical training to develop procedural skills, with evaluations of the haptic trainers generally showing noninferiority in skills acquisition [4]. In particular, haptic trainers are valued for developing psychomotor skills in surgical novices [5]. Similarly, outside the surgical specialties, a study comparing haptic trainers and mannequins for teaching central line placement to medical students found that haptic trainers increased medical students’ comfort in adapting their technique to accommodate different patient anatomies [6].

We evaluated haptic-augmented SBS for emergency medicine procedures, specifically lateral canthotomy (LC) and needle decompression (ND). Our primary objective was to compare the two modalities in terms of user experience, specifically regarding realism, ease of use, and educational utility. Our secondary objective was to evaluate procedural knowledge acquisition among emergency medicine learners using both haptic-augmented and traditional simulation methods. We hypothesized that the haptic simulators would demonstrate equivalence in knowledge acquisition and be preferred by learners over traditional task trainers.

## Materials and methods

Study design and setting

This research was conducted at a medical school simulation center in the United States. Approval was granted by the university's institutional review board for exempt status, without the need for written informed consent. Verbal consent was obtained from all participants.

The study design involved a comparison of task trainer-based simulation (control group) with SBS featuring haptic feedback (experimental group) for two different emergency medicine procedures: LC and ND.

The haptic trainer used was a commercially available product (3D Systems Touch^TM^; 3D Systems, Inc., Rock Hill, SC, USA) with custom programming built with Unity^TM^ (Unity Technologies; San Francisco, CA, USA). The trainer operated as a motorized, pen-like stylus connected to a grounded robotic arm. It read user input across six degrees of freedom, tracking translational movements across X, Y, and Z axes, alongside rotational movements including pitch, yaw, and roll while supplying active force feedback output across three degrees of freedom [7]. The haptic interface utilized a single-handed stylus to control all virtual instruments. The stylus was used to position the selected instrument, such as scissors or forceps. Buttons on the pen triggered the instruments’ actions, such as the closure of the scissors. Although clinical procedures typically require two hands, the software allowed for sequential instrument selection and movement via the single device. A custom-made task trainer was used for LC [8], whereas ND was performed on a Laerdal SimMan 3G Trauma^TM ^(Laerdal Medical, Stavanger, Norway). On a given day, participants completed a baseline pre-test about their existing experiences of learning about and performing the procedure. Participants were then divided in alternating order into an experimental group featuring the haptic trainer and a control group featuring the traditional task trainer.

Participants assigned to the haptic trainer received a brief overview of the instruments required for the procedure within the SBS custom programming. The software then guided participants through each step, with visual cues to indicate the appropriate positioning of instrumentation available if participants became stuck. Participants could not advance past a stage until they had adequately positioned and performed the necessary motions using the haptic stylus.

Participants in the control group received a proctor-led, step-by-step walkthrough on each model. After completing their initial assigned group, participants rotated to the opposite group and performed the procedure using the other method. Upon completion of both groups, participants completed a post-test composed of the same knowledge questions as the pre-test, as well as survey questions about their experiences with both training methods.

Study subjects

**Figure 1:**
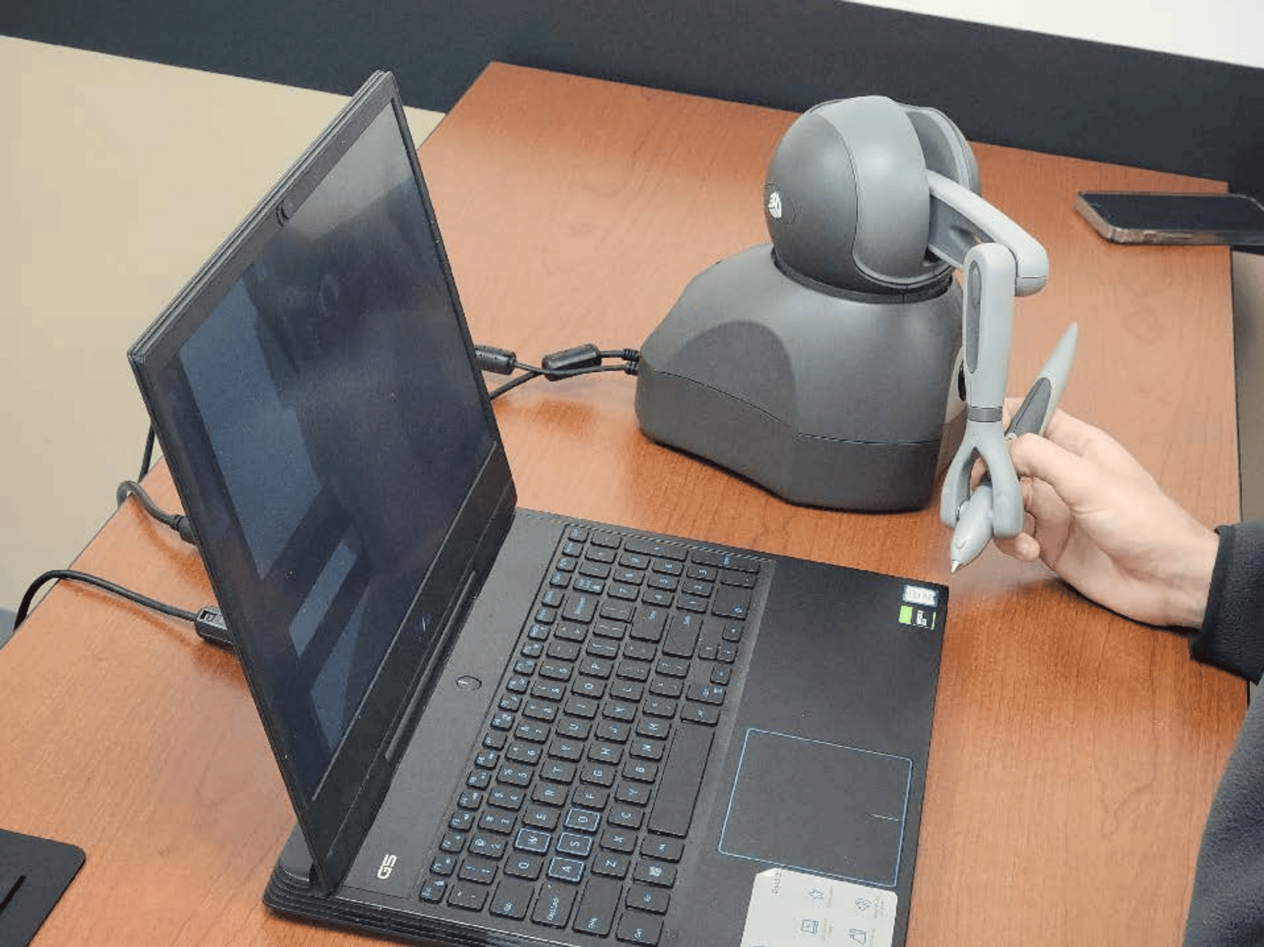
Haptic stylus and laptop

**Figure 2:**
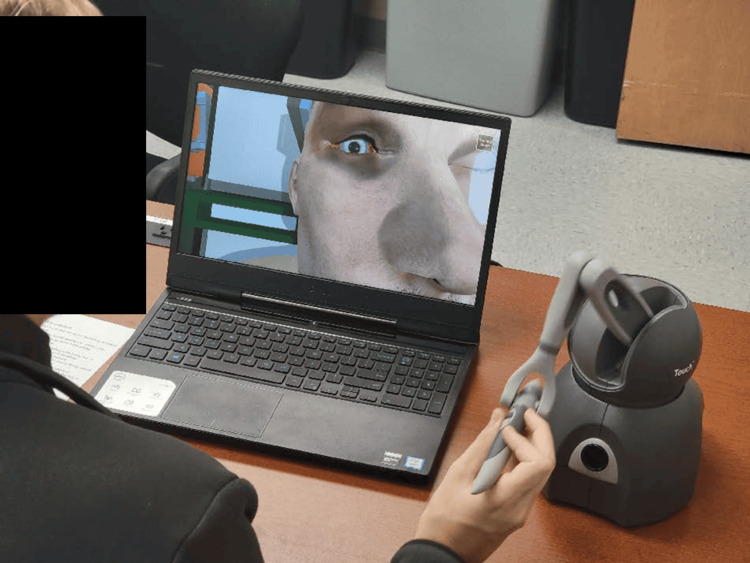
Lateral canthotomy by a haptic trainer

**Figure 3:**
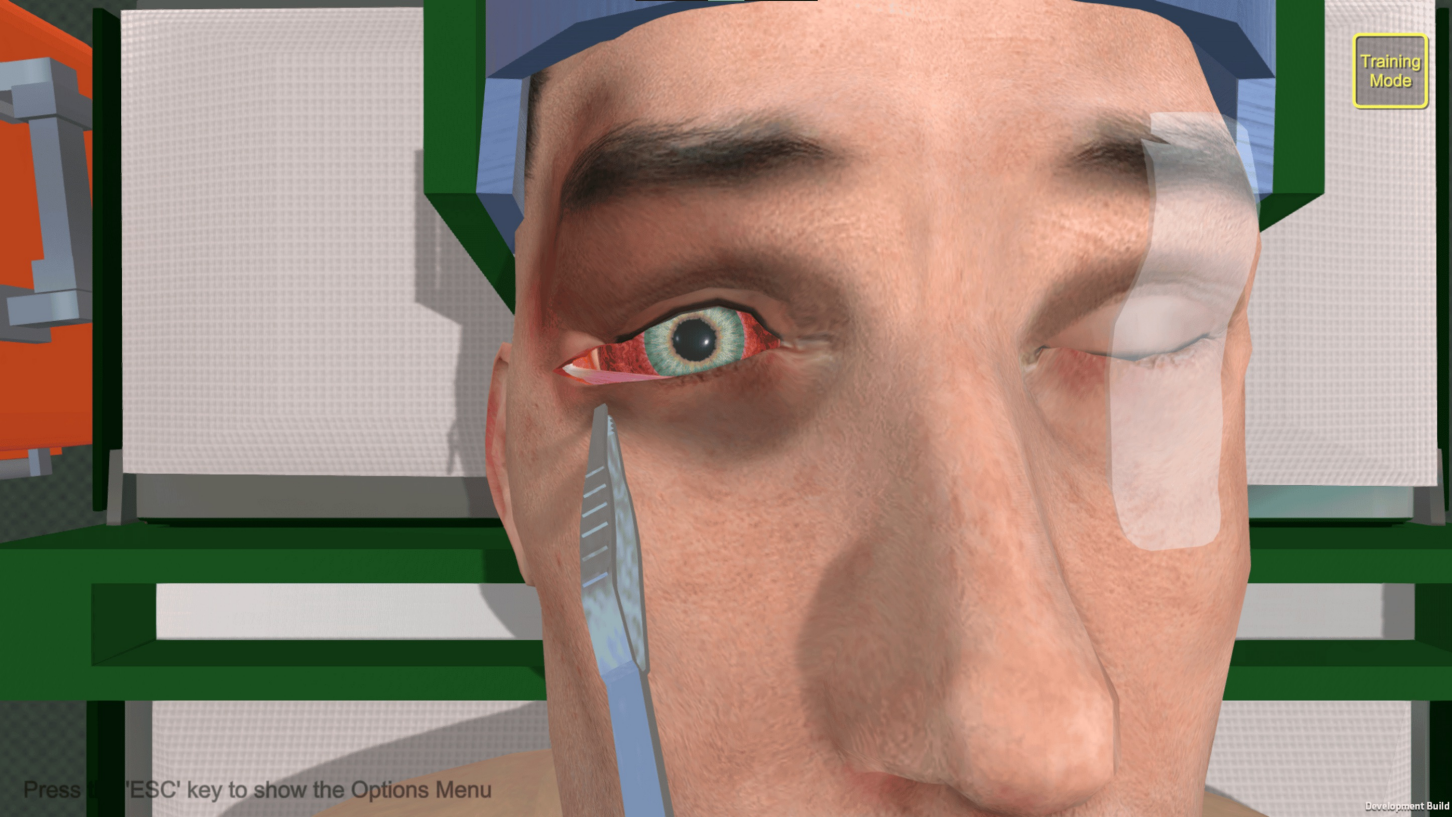
Lateral canthotomy software

**Figure 4:**
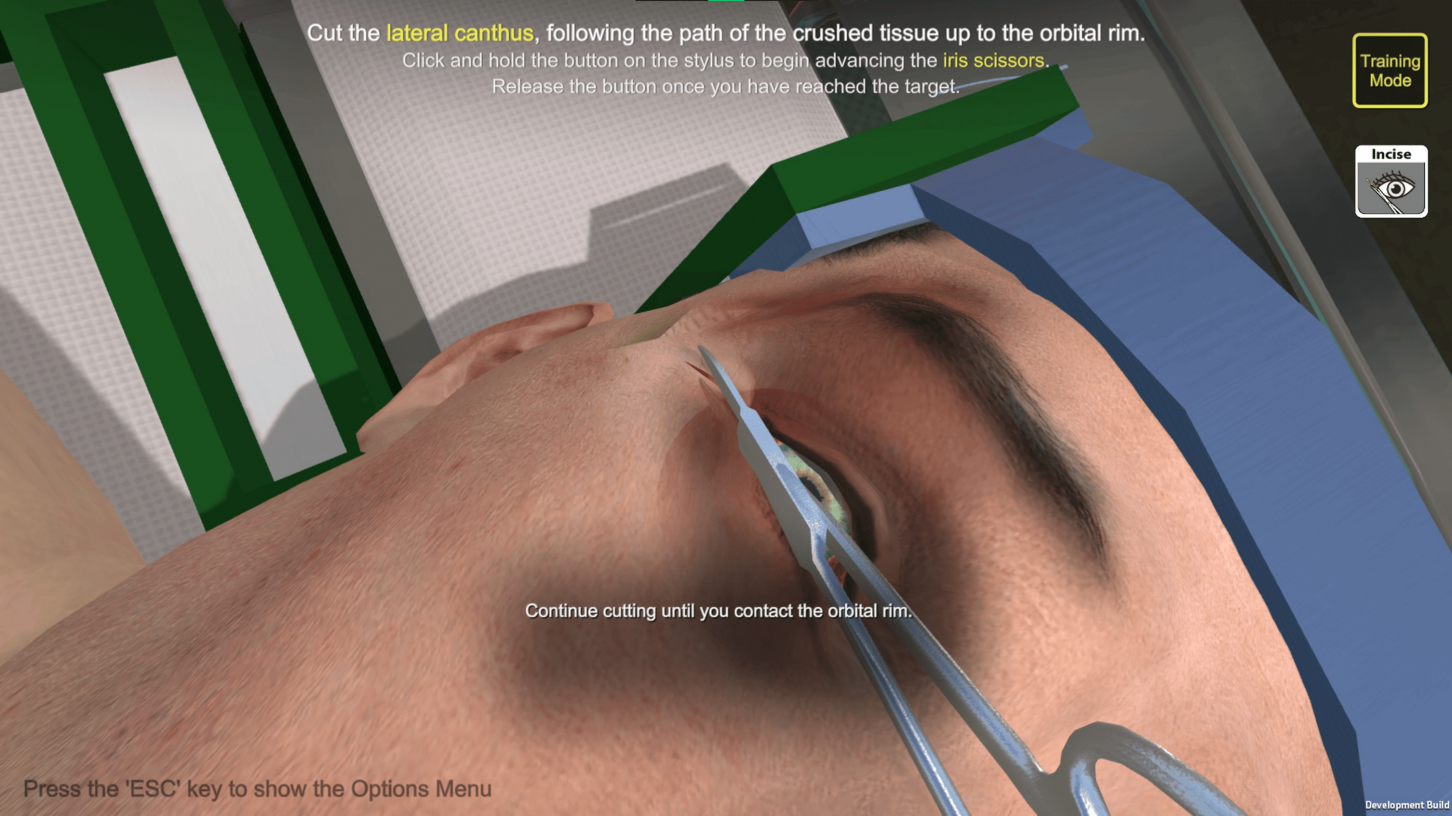
Guided dissection of the lateral canthus

**Figure 5:**
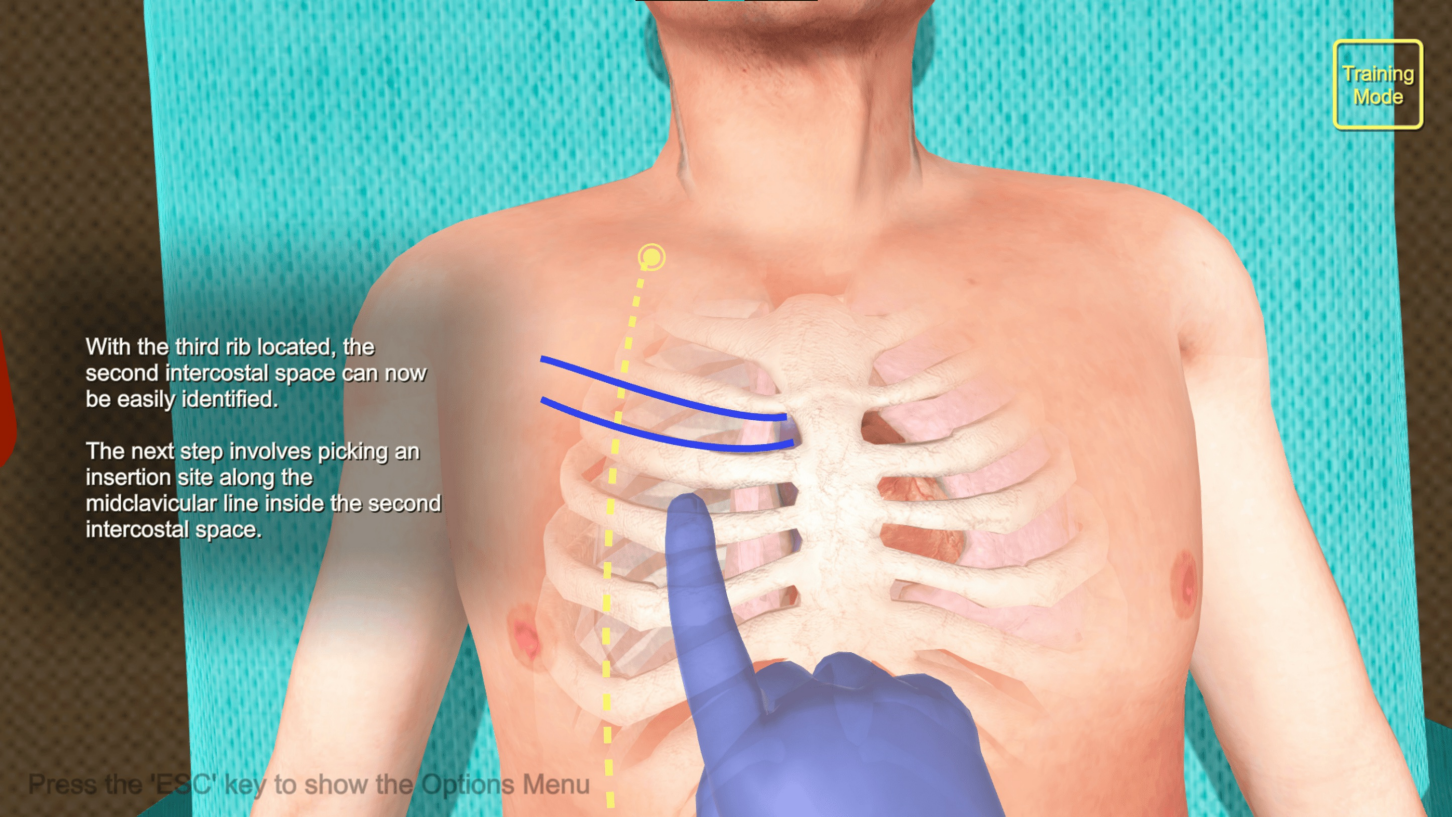
Needle decompression software

Participants (n* *= 22) consisted of 14 emergency medicine residents (postgraduate year (PGY)-1 to PGY-3) and eight medical students (MS-2 and MS-4). They were recruited through announcements during educational conferences, emails, and direct interactions. Participants were made aware that the study was completely independent from their educational curricula and would not impact their grades or evaluations, and that they could withdraw from the study at any time without penalty. De-identified images of the participants using the traditional and haptic models, as well as screenshots from the software, are available in Figures 1-5.

Measurements

All participants completed pre- and post-simulation knowledge assessments composed of five multiple-choice questions for both procedures regarding indications, contraindications, and basic skills. The accuracy of participants' responses was compared between pre- and post-simulation assessments using the paired-samples proportion Newcombe test to determine confidence intervals and the McNemar test for one-sided significance.

Participants additionally completed surveys composed of questions assessing their prior familiarity with the procedures and their overall clinical experience level, as well as 12 statements graded on a Likert scale. The following values were assigned for the Likert scale responses: Strongly agree (5), agree (4), neutral (3), disagree (2), strongly disagree (1). The median of each response was calculated along with the interquartile range.

## Results

Table 1 summarizes the baseline characteristics of the 22 participants, consisting of 14 residents and eight medical students. Clinical experience on real patients was minimal across the cohort, with an average of 0.09 procedures for LC and 0.14 for ND. Baseline confidence for the total group was 1.5 (IQR, 1-3) for LC and 2.0 (IQR, 1-3) for ND. Residents reported higher median confidence levels (2.5 for LC and 3.0 for ND) compared to medical students, who reported a median confidence of 1.0 for both procedures.

**Table 1:** Prior experience and confidence of learners (n = 22) LC: lateral canthotomy; ND: needle decompression; IQR: interquartile range. Resident count for ND reflects n = 12 due to an incomplete survey response in the raw data. The asterisk (*) denotes that the resident count for the needle decompression (ND) row (n=12 instead of the full n=14) because of an incomplete survey response in the raw data.

	Residents (n* *= 14)	Medical Students (n = 8)	Total (n* *= 22)
Prior simulation training, n (%)	10 (71.4)	1 (12.5)	11 (50.0)
Real patient experience (LC)*, *n (%)	2 (14.3)	0 (0.0)	2 (9.1)
Real patient experience (ND), n (%)	3 (25.0)*	0 (0.0)	3 (13.6)
Confidence (LC), median (IQR)	2.5 (1.25-3.0)	1.0 (1.0-1.0)	1.5 (1.0-3.0)
Confidence (ND), median (IQR)	3.0 (2.75 to 4.0)	1.0 (1.0 to 1.0)	2.0 (1.0 to 3.0)

Knowledge assessments showed significant improvement in scores on all five questions in the LC simulation, with an average score increase of 20.9% (p < 0.001, Table 2). Knowledge assessments in the ND simulation showed statistically significant score increases on questions regarding needle gauge choice (p = 0.007, Table 3) but significant decreases on questions regarding anatomic sites with the lowest failure rates (p = 0.004, Table 3). No significant differences were noted in questions regarding variations in chest wall thickness at different anatomic sites, ideal needle length, or diagnostic indications (p = 0.500 and 0.090).

**Table 2:** Lateral canthotomy pre- and post-simulation knowledge assessments (n = 22) Individual Qs were calculated using paired-samples proportion Newcombe's test for confidence intervals and McNemar's test for one-sided significance. Total was calculated using a paired-sample *t*-test.

	Pre-test Percentage Correction	Post-test Percentage Correction	Difference (95% CI)	p-Value
Q1 Which of the following is an indication for lateral canthotomy?	86.4%	100%	13.6% (-3.7-33.3)	0.042
Q2 How should a patient be positioned during lateral canthotomy?	59.1%	95.5%	36.4% (12.2-56.9)	0.002
Q3 In which direction should the cut be made when performing cantholysis?	31.8%	63.6%	31.8% (9.9-49.2)	0.004
Q4 At which intraocular pressure is lateral canthotomy indicated?	63.6%	86.4%	22.7% (-1.6-44.3)	0.029
Q5 Which of the following is a contraindication for lateral canthotomy?	95.5%	95.5%	0% (-17.8-17.8)	0.500
Total	67.3%	88.2%	20.9% (9.8-32.0)	<0.001

**Table 3:** Needle decompression pre- and post-simulation knowledge assessments (n = 22) Individual Qs were calculated using paired-samples proportion Newcombe's test for confidence intervals and McNemar's test for one-sided significance. Total was calculated using a paired-sample *t*-test. CI: confidence interval

	Pre-test Percentage Correction	Post-test Percentage Correction	Difference (95% CI)	p-Value
Q1 True or false? Needle decompression has better outcomes when a 5 cm needle is placed versus an 8 cm needle at the second rib space at the anterior axillary line.	55.6%	55.6%	0% (-22.2-22.2)	0.500
Q2 Which gauge needle is best for needle decompression?	66.7%	100%	33.3% (8.8-56.3)	0.007
Q3 Which diagnosis indicates the need for needle decompression?	100%	100%	0% (-17.6-17.6)	-
Q4 At which site does the needle decompression have the lowest failure rate?	44.4%	5.6%	-38.9% (-61.1- -10.9)	0.004
Q5 True or false? On average, the chest wall is 1 cm thicker at the second intercostal space compared to the fifth.	77.8%	61.1%	-16.7% (-39.6-8.9)	0.090
Total	68.9%	64.4%	4.4% (-13.8-4.9)	0.166

During the post-simulation evaluation using a Likert scale, participants rated the haptic trainer for LC with median scores of 4 to 5 for improved realism, enjoyability, ease of use, utility, and novelty compared to the standard model (Table 4). For ND, the haptic trainer received median scores of 3 for improved realism, enjoyability, and preference compared to the standard TraumaMan^TM^ model. Statements regarding the haptic trainer’s ease of use, utility in identifying anatomic landmarks, interactivity, and overall desirability to incorporate the tool into medical training received scores of 4 or 5 (Table 5).

**Table 4:** Lateral canthotomy post-simulation opinion questions (n = 22) MR: mixed reality; IQR: interquartile range.

	Median Likert Score (IQR)
Q1 The MR simulation was more realistic than the standard model.	4 (3.75-5)
Q2 The MR simulation was more enjoyable than the standard model.	4 (3-5)
Q3 For future procedure-based simulations, MR is preferred over the standard model.	4 (3-5)
Q4 The MR technology helped identify anatomical landmarks.	4.5 (4-5)
Q5 The MR technology was easy to use.	4 (3-5)
Q6 The MR technology was enjoyable to use.	5 (4-5)
Q7 The MR technology will improve my lateral canthotomy skills.	4.5 (4-5)
Q8 The MR technology offered features that support learning.	5 (4-5)
Q9 The MR technology incorporates interactivity to hold attention and promote learning.	5 (4-5)
Q10 The MR technology incorporated novel features that promoted learning.	5 (4-5)
Q11 MR technology could be a useful tool in my medical procedures skill training.	5 (4-5)
Q12 Incorporation of MR technology into medical training would be useful.	5 (4-5)

**Table 5:** Needle decompression post-simulation opinion questions (n = 22) MR: mixed reality; IQR: interquartile range.

	Median Likert Score (IQR)
Q1 The MR simulation was more realistic than the TraumaMan model.	3 (3-4)
Q2 The MR simulation was more enjoyable than the TraumaMan model.	3 (3-5)
Q3 For future procedure-based simulations, MR is preferred over the TraumaMan model.	3 (2.5-4)
Q4 The MR technology helped identify anatomical landmarks.	4 (4-5)
Q5 The MR technology was easy to use.	4 (4-5)
Q6 The MR technology was enjoyable to use.	5 (4-5)
Q7 The MR technology will improve my needle decompression skills.	4 (4-5)
Q8 The MR technology offered features that support learning.	5 (4-5)
Q9 The MR technology incorporates interactivity to hold attention and promote learning.	5 (4-5)
Q10 The MR technology incorporated novel features that promoted learning.	5 (4-5)
Q11 MR technology could be a useful tool in my medical procedure skill training.	5 (4-5)
Q12 Incorporation of MR technology into medical training would be useful.	5 (4-5)

## Discussion

This study evaluated the efficacy of haptic training simulators in enhancing skill acquisition of emergency procedures, specifically LC and ND, compared to traditional task trainers. Our results suggest that haptic simulators can offer significant educational benefits, particularly in procedures such as LC that require feeling the tissue and detailed hand movements. Participants demonstrated significant improvement in knowledge scores following the LC simulation with haptic feedback. The enhanced realism and interactive elements of the haptic trainer appeared to foster greater confidence and skill retention and were broadly enjoyed by learners who considered it a significant improvement over traditional task trainers, as reflected by post-simulation surveys.

The haptic trainer's performance in ND was less compelling. Participants expressed only moderate satisfaction with the realism of the haptic trainer in ND, possibly reflecting the simpler and more tactile nature of the procedure itself, which may not be as well-suited for haptic simulation as more complex procedures. This would align with findings in a systematic review of haptic feedback trainers in teaching laparoscopic surgical skills; the review concluded that adding haptic feedback was most beneficial for complex tasks in which it appeared to shorten the learning curve [9]. Other studies of haptic simulators in teaching ND have also suggested the haptic styli of these particular trainers may not effectively mimic the multi-fingered action of palpating rib spaces and the force of driving a needle through intercostal muscles and tissue [10]. It may also be due to an increase in the baseline learner's familiarity with ND compared with LC. Moreover, when compared to a commercial simulator such as SimMan, the haptic trainer likely offers only a modest quality improvement, whereas its advantages stand out more clearly against a custom-built trainer.

The significant decrease in scores for anatomic site selection in ND was characterized by a shift from correct or varied answers to the second intercostal space (ICS). Although the second ICS was once the standard, the study software emphasized the fifth ICS at the anterior axillary line as having the lowest failure rates. Several learners likely defaulted to the second ICS due to historical teaching, despite the haptic simulation and modern guidelines advocating for the fifth ICS [11]. This suggests that for procedures with deeply ingrained but outdated practices, a single simulation session may not be sufficient to override established clinical heuristics.

The haptic trainers provide a high cost and logistical advantage over traditional task trainers by allowing repeated practice without consumable materials, such as silicone skins, which are required for each student on physical models. This makes them particularly appealing in resource-limited settings or for training large groups. In general, virtual-reality simulation tools often have a relatively high initial cost compared to traditional simulators, but lower maintenance costs [12].

Furthermore, the software can be easily programmed to track individual learners' progress and can integrate with learning management systems, offering built-in assessments to monitor learners’ progress and scores. In addition, it has the potential for providing more objective feedback on measures, such as angle and orientation of instruments, proximity to neighboring anatomic structures, and other fine data that may not be attainable on a traditional task trainer. These metrics themselves may be indicators of procedural expertise, with research demonstrating consistent differences in metrics, such as time to completion and deviation of needle tip from an ideal path, when comparing attending physicians and surgical interns in a haptic central line trainer [13]. They have also shown promise in improving patient-centered outcomes. For example, the presence of force feedback automatically reduced the user's plunge depth by an average of 33 mm in studies evaluating haptic feedback during simulated orthopedic bone drilling, thereby significantly lowering the risk of catastrophic neurovascular injury upon breaching the far cortex of the bone. Haptic simulators provide objective, automated, and immediate feedback by establishing algorithmic thresholds based on normative expert performance [14]. Such metrics could also prove useful in, for example, the development of a mastery learning curriculum. A comparison of haptic trainers with this kind of feedback and traditional task trainers in central line education among first-year surgical residents also suggested that this kind of feedback may help learners develop more accurate appraisals of their procedural skills and confidence [15].

Qualitative learner feedback highlighted specific technical limitations regarding the haptic interface. Although the simulator was rated highly for utility, participants noted that the haptic stylus constrained the dexterity and complex hand movements required for these procedures in a clinical setting. Several learners advocated for a bimanual (two-handed) configuration to better replicate real-world procedural ergonomics. Additionally, a small subset of participants experienced difficulty orienting the haptic stylus within the sensor's physical field of view, occasionally leading to a misalignment between their hand movements and the on-screen instrumentation. These observations suggest that although haptic technology is a valuable adjunct, future iterations should prioritize bimanual feedback and a more intuitive spatial mapping to enhance procedural fidelity.

Limitations

This study has several limitations. First, the sample size was relatively small, with only 22 participants, limiting the generalizability of the findings. Future studies with larger cohorts are needed to confirm these results. Second, the study was conducted at a single academic center, which may not reflect the diversity of training experiences across other institutions. Because knowledge tests were administered only at the beginning and end of the study, they reflect the combined impact of both training methods. This prevents isolating the specific knowledge acquisition attributable solely to the haptic simulator. Additionally, the custom-built nature of the LC task trainer may have contributed to the increased satisfaction scores with the haptic trainer, as the task trainer may not have offered the same fidelity as commercially developed trainers. This makes it more difficult to directly compare the two training methods. Moreover, although the haptic trainers allowed for repetitive practice without the need for consumable materials, the study did not directly assess the long-term impact of this ease of repeated practice on skill retention. An additional difference between the haptic simulator and traditional training is the reliance on a single-handed interface. Future iterations could incorporate a second haptic device to better simulate bimanual coordination and improve procedural fidelity. Finally, the haptic trainer software was custom-built, which may limit reproducibility in settings without similar technological resources.

## Conclusions

Haptic simulators may be a useful simulation adjunct for enhancing procedural skills in emergency medicine. In this small cohort, haptic trainers were rated highly for procedures requiring fine motor control, such as LC. However, given the pilot nature of this study and the identified confounding factors, these results should be viewed as a proof of concept for digital haptic integration in medical education. Their ability to allow repeated practice without consumable materials offers high cost and logistical benefits, particularly in resource-limited settings.
